# Risperidone in situ microimplant(Okedi®): a ten-months observational study in the Department of Mental Health of Rimini (Italy)

**DOI:** 10.1192/j.eurpsy.2026.11769

**Published:** 2026-07-31

**Authors:** M. P. Rapagnani, L. Veronesi, F. Sartini

**Affiliations:** Psychiatry, Ausl della Romagna, Rimini, Italy

## Abstract

**Introduction:**

Risperidone In Situ Microimplants (ISM®) is a new intramuscular (IM) LAI formulation providing plasma levels in the therapeutic range within the first hours after administration which are maintained for up to one month without the need for oral risperidone supplementation or loading doses.The ISM technology enables the extended delivery of compounds with the following advantages: less variability, enhanced stability, rapid reconstitution, and straightforward injection, making it uncomplicated for the patient and provider to follow the prescribed treatment.

**Objectives:**

The aim of the study is to evaluate the efficacy, safety and tolerability of Risperidone ISM® in patients with psychotic disorders in a real world use.

**Methods:**

We retrospectively reviewed medical records of patients, from October 2024 (first patient to have the risperidone ISM therapy) to August 2025, inpatients ed outpatients, that have more than 18 years. The study is real-world without exclusion criteria. We valuated retrospectively the days of hospitalization after the administration, the number of emergency room accesses (ER), the tolerability and the efficacy analyzing the “repository of AUSL della Romagna”, the discharge letter from the Psychiatric Hospital of Rimini and the “CURE” program of the Psychiatric Service of Rimini.

**Results:**

From October 2024 to August 2025 we enrolled 10 patients, 7 males and 2 females. Average age 49,7. Four patients started the therapy in Psychiatric Ward, others as outpatients. The dosage are: 5 patients 100 mg and 4 patients 75 mg. The diagnosis are: 3 delusional disorder, 1 bipolar disorder, 1 schizoaffective disorder, 4 schizophrenia. Two patients received the Risperidone ISM from another LAI. Others therapies: mood stabilizers, other antipsychotics, lithium. 1 pt reported hyperprolactinemia. No other side effects reported. One pt had an hospitalitzation after the first injection, but very close to the discharge, another pt had an hospizalization but is a very severe and chronic bypolar disorder and 1 pt had an access in the ER.

**Conclusions:**

Risperidone ISM® is a new monthly LAI antipsychotic that provides immediate and sustained drug plasma levels without loading doses or oral supplementation. Risperidone ISM®, using monthly intramuscular doses of 75 mg or 100 mg, was well tolerated, and provided significant improvement of the symptomatology and disease severity in acutely exacerbated patients with schizophrenia.an effective, safe, and well tolerated monthly antipsychotic for the long-term treatment of schizophrenia in adults, regardless of the initial disease severity or whether patients were previously treated with Risperidone ISM® during an acute exacerbation or switched from oral risperidone in a stable disease setting. Intramuscular risperidone once monthly was an effective well tolerated antipsychotic.

**Disclosure of Interest:**

None Declared

